# The deubiquitylase USP7 is a novel cyclin F-interacting protein and regulates cyclin F protein stability

**DOI:** 10.18632/aging.204372

**Published:** 2022-11-05

**Authors:** Savitha S. Sharma, W. Jack Pledger, Paturu Kondaiah

**Affiliations:** 1 Department of Molecular Reproduction, Development and Genetics, Indian Institute of Science, Bengaluru, 560012, India; 2 Sri Shankara Cancer Hospital and Research Centre, Bengaluru, 560004, India; 3 Department of Surgery, University of Utah Health, Huntsman Cancer Institute, Salt Lake City, UT 84132, USA

**Keywords:** cyclin F, atypical cyclins, USP7, cell cycle, genomic integrity

## Abstract

Cyclin F, unlike canonical and transcriptional cyclins, does not bind or activate any cyclin-dependent kinases. Instead, it harbors an F-box motif and primarily functions as the substrate recognition subunit of the Skp1-Cul1-F-box E3 ubiquitin ligase complex, SCF^Cyclin^ ^F^. By targeting specific proteins for ubiquitin-mediated proteasomal degradation, cyclin F plays a critical role in the regulation of centrosomal duplication, DNA replication and repair, and maintenance of genomic stability. Cyclin F abundance and activity are tightly regulated throughout the cell cycle. However, the molecular mechanisms regulating cyclin F are scantily understood. Here, we identify the deubiquitylase USP7 as a novel cyclin F-interacting protein. We observe that USP7 stabilizes cyclin F protein and that this function is independent of the deubiquitylase activity of USP7. Additionally, our data suggest that USP7 is also involved in the regulation of cyclin F mRNA. Pharmacological inhibition of the deubiquitylase activity of USP7 resulted in downregulation of cyclin F mRNA.

## INTRODUCTION

Orderly progression through the cell cycle is driven by the periodic oscillations in the activity of cyclin-dependent kinases (CDKs) [1]. The activity of CDKs, in turn, is controlled by their binding to allosteric regulatory proteins called cyclins. There are at least 30 members in the cyclin family; while many of these contribute to various aspects of cell-cycle control, not all are known to be CDK activators [2]. Among the cyclins that play a crucial role in cell-cycle progression, is cyclin F. It is most similar to cyclin A, both in terms of amino acid sequence and the cyclic pattern of expression during the cell cycle [3]. However, unlike cyclin A and many other cyclins, cyclin F does not bind or activate CDKs [3]. Instead, cyclin F is the founding member of the F-box family of proteins, whose 69 members share a conserved F-box domain [4]. Using its F-box, cyclin F interacts with Skp1, which simultaneously recruits Cul1 (and RBX1 with Cul1): together they assemble into a functional SCF^Cyclin F^ (Skp1-Cul1-F box) E3 ubiquitin ligase complex [4, 5]. Within this complex, cyclin F functions as the substrate-recognition subunit and targets specific proteins for ubiquitylation, and subsequent degradation [5].

Among the cyclin F substrates identified so far are CP110, NuSAP1, RRM2, CDC6, SLBP, RBPJ, activator E2Fs, and E2F7 [6–13]. Additionally, other interaction partners that modulate the function of, or are themselves modulated by cyclin F have also been identified; which include b-Myb, p27^Kip1^, Akt, Casein kinase II, β-TrCP, and VCP [14–18]. Cyclin F functionally interacts with these substrates and interaction partners to chiefly regulate genomic and chromosomal stability. Of note, experimental depletion of cyclin F leads to multiple deleterious outcomes: centrosomal overduplication and micronucleation (through stabilization of centrosomal protein, CP110) [6, 15], unbalanced dNTP pools and increased rate of mutation (due to accumulation of ribonucleotide reductase family member 2; RRM2) [8], failure to maintain ionizing-radiation-induced G2-phase arrest and premature entry into mitosis (through enhanced cyclin A2-CDK2−mediated phosphorylation of b-Myb) [14], DNA re-replication (due to accumulation of CDC6) [9], persistent increase in H2A.X levels and signaling after DNA damage (due to stabilization of SLBP) [10], and enhanced expression of E2F-target genes combined with accelerated G1/S transition (due to stabilization of activator E2Fs) [12]. Likewise, mutations in cyclin F, observed in patients with amyotrophic lateral sclerosis and frontotemporal dementia, cause abnormal ubiquitylation and accumulation of target proteins, including that of RRM2 and TDP-43 [19].

Given this role of cyclin F in maintaining protein homeostasis and genomic integrity, it is likely that cyclin F is a tightly regulated protein. However, the mechanisms regulating cyclin F are scantily understood. As mentioned earlier, the mRNA and protein levels of cyclin F oscillate throughout the cell cycle [3]. The protein levels of cyclin F begin to accumulate in S phase, peak in G2, and are undetectable in mitosis and G1 [3]. Further, cyclin F is a short-lived protein, with a half-life of less than 1 hour [3, 20]. Although, initially it was suggested that cyclin F undergoes a metalloprotease-mediated proteolysis [20], more recent evidence indicate that it is also targeted by multiple E3 ubiquitin ligases for proteasomal degradation: APC/C^Cdh1^ in G1 phase, SCF^βTrCP^ during M phase, and an as yet unidentified, ATR-dependent E3 ubiquitin ligase in response to DNA damage [8, 18, 21].

In this study, we identify the deubiquitylase USP7 as a novel cyclin F-interacting protein. We demonstrate that USP7 stabilizes cyclin F protein and that this function is independent of the deubiquitylase activity of USP7. Additionally, our data suggest that USP7 is also involved in the regulation of cyclin F mRNA.

## RESULTS

### Cyclin F associates with USP7

**Figure 1:**
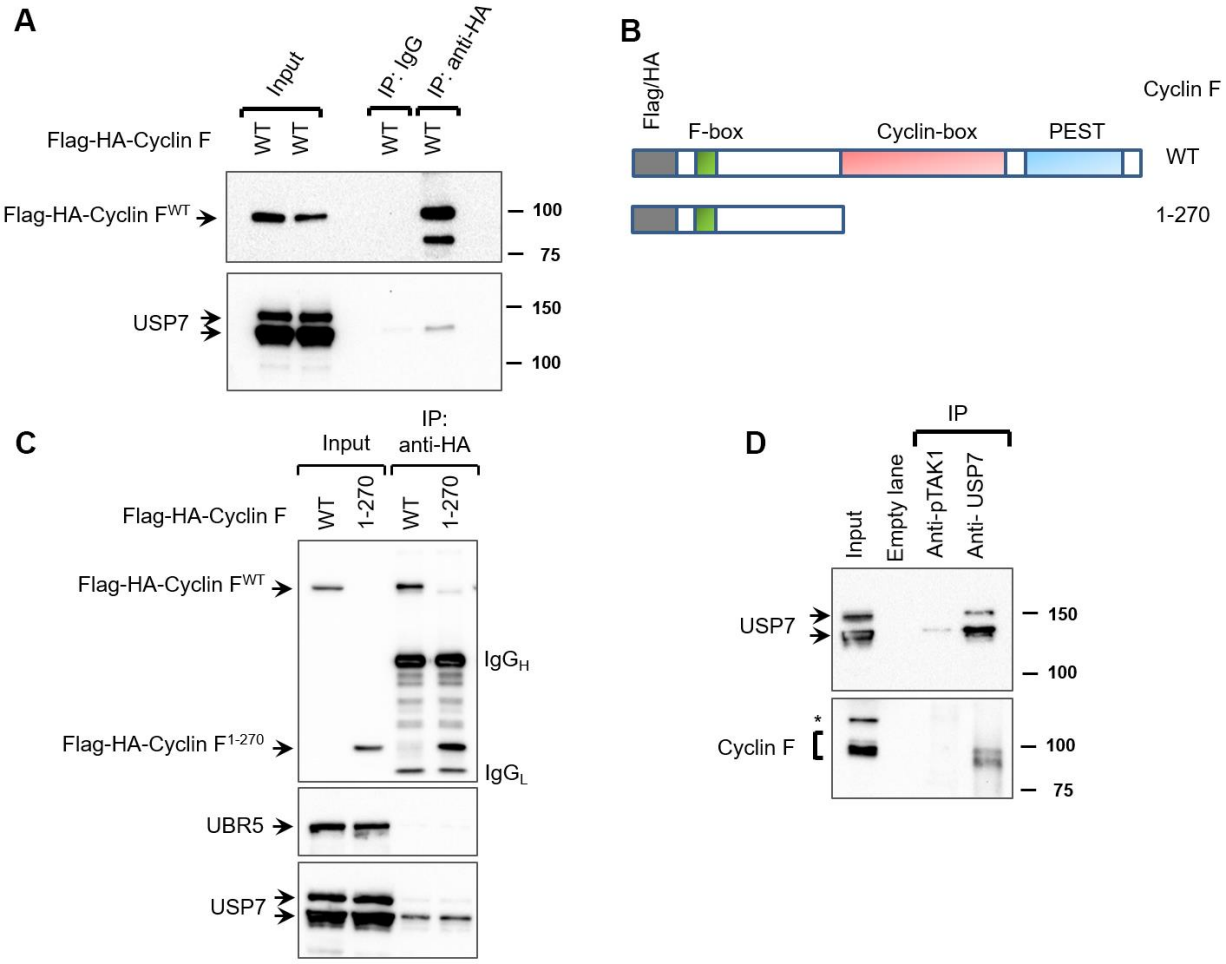
**Cyclin F interacts with USP7.** (**A**) HEK-293T cells transfected with Flag-HA-Cyclin F were lysed and immunoprecipitated with anti-HA antibody or non-specific mouse immunoglobulin (IgG) as loading control. Immunocomplexes were immunoblotted as indicated. (**B**) Schematic representation of cyclin F WT and cyclin F^1-270^ truncated mutant, highlighting the F-box, cyclin-box and PEST regions. (**C**) HEK-293T cells transfected with Flag-HA-Cyclin F WT or Flag-HA-Cyclin F^1-270^ were immunoprecipitated and immunoblotted as indicated. (**D**) Endogenous USP7 was immunoprecipitated from HEK-293T cell extracts, with anti-USP7 antibody or an unrelated, anti-p-TAK1 antibody as a loading control (denoted as control). Immunocomplexes were immunoblotted as indicated. Asterisk denotes non-specific band.

To discover novel cyclin F-interacting proteins, we had previously performed mass spectrometry and identified proteins present in anti-HA immunoprecipitates from extracts of the U2OS human osteosarcoma cell line transiently expressing Flag-HA-Cyclin F or empty vector (Supplementary File 1 and Supplementary Information). Such proteins included known cyclin F-interacting proteins (for example, RRM2, CDC6, and casein kinase II). They also included USP7 (ubiquitin-specific protease 7), whose association with cyclin F has not been previously reported. USP7 is a deubiquitylase, and a master regulator of pathways maintaining genomic integrity: one such well-characterized function is its regulation of the MDM2-p53 axis [22]. To validate the interaction between cyclin F and USP7, we first transiently expressed Flag-HA-Cyclin F in HEK-293T cells. Immunoprecipitation of the ectopic cyclin F with antibodies against the HA tag, resulted in coprecipitation of the endogenous USP7 (Figure 1A). Normal mouse IgG did not coprecipitate Flag-HA-Cyclin F or USP7 (Figure 1A). These results suggest that cyclin F specifically interacts with USP7. The cyclin-box of cyclin F has been shown to mediate interaction with a number of its substrates (Figure 1B). To examine whether the cyclin-box mediated its interaction with USP7, we made use of the truncation mutant, Flag-HA-Cyclin F^1-270^ (lacking both the cyclin-box and PEST region; Figure 1B). Like the wild-type Flag-HA-Cyclin F, Flag-HA-Cyclin F^1-270^ was still able to coprecipitate endogenous USP7, suggesting that the interaction of cyclin F and USP7 is independent of the cyclin-box or PEST region of cyclin F (Figure 1C). Further, in this experiment, while USP7 coprecipitated with transiently expressed Flag-HA-Cyclin F, UBR5 (an E3 ubiquitin ligase) did not (Figure 1C).

We next assessed whether endogenous cyclin F interacts with endogenous USP7. Since the commercially available antibodies against cyclin F were unsuitable for immunoprecipitation, we chose to instead immunoprecipitate endogenous USP7 and check for coprecipitation of cyclin F. In HEK-293T cells, cyclin F was detected in USP7 immunoprecipitates, suggesting that endogenous cyclin F interacts with endogenous USP7 (Figure 1D). Importantly, mock immunoprecipitation with an unrelated antibody (anti-p-TAK1) did not appreciably detect USP7 or cyclin F (Figure 1D).

### SCF^Cyclin F^ does not regulate USP7 protein levels

**Figure 2:**
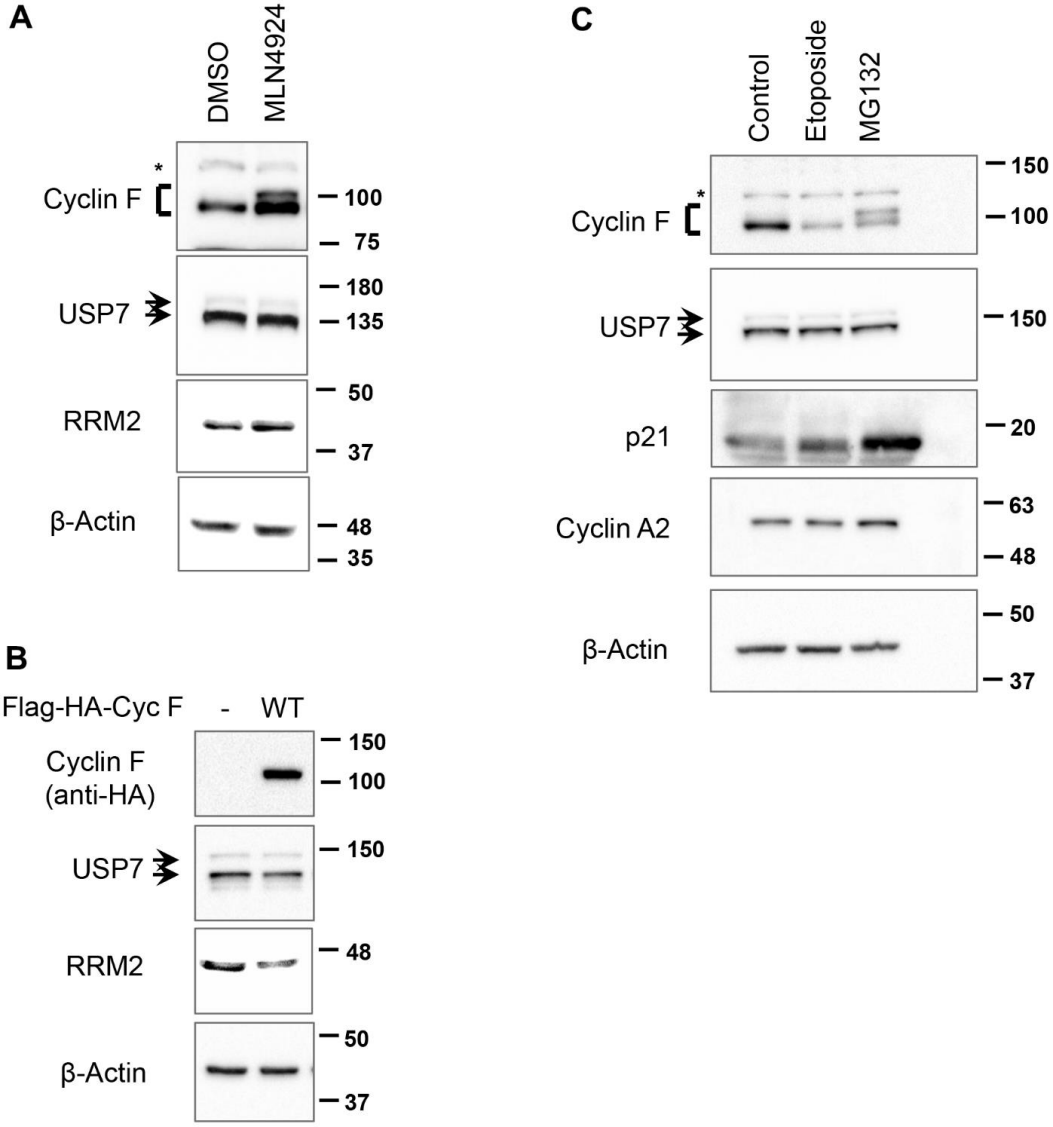
**SCF^Cyclin F^ does not regulate USP7 protein levels.** (**A**) HCT116 cells treated with either DMSO or MLN4924 (3 μM) for 5 h were lysed, and immunoblotted as indicated. β-Actin was the loading control. Asterisk denotes non-specific band. (**B**) HEK-293T cells were transfected with an empty vector or Flag-HA-Cyclin F for 48 h, lysed, and immunoblotted as indicated. (**C**) HeLa cells treated with DMSO, etoposide (10 μM) or MG132 (10 μM) for 4 h were lysed, and immunoblotted as indicated. Asterisk denotes non-specific band.

SCF^Cyclin F^ is an ubiquitin ligase, while USP7 is a deubiquitylase. To test whether SCF^Cyclin F^ targets USP7 for ubiquitylation and subsequent degradation, we first inhibited all SCF ligases with MLN4924, an inhibitor of NAE (NEDD8-activating enzyme); the activity of the latter is required for the activity of SCF ligases [23]. We found that the cyclin F levels are increased by treatment with MNL4924 compared to DMSO-treated controls (Figure 2A), suggesting that cyclin F could be regulated by an autocatalytic mechanism or by a different F-box protein. Additionally, in MNL4924-treated cells we observed a slower-migrating band detected specifically by anti-cyclin F antibody (Figure 2A, lane 2); we speculate this to be phosphorylated cyclin F, accumulating along with the non-phosphorylated form in the absence of cyclin F ubiquitylation [16]. On the other hand, treatment with MLN4924 increased RRM2, a well-documented cyclin F substrate, but did not affect USP7 protein levels, suggesting that SCF ligases, including SCF^Cyclin F^, do not regulate USP7 protein abundance (Figure 2A).

In a variety of cell lines, cyclin F overexpression has been shown to downregulate a number of its substrates, including CP110 and RRM2, by ubiquitin-mediated proteasomal degradation [6, 8]. In line with these reported findings, overexpression of cyclin F in HEK-293T cells led to a decrease in RRM2 levels, but did not affect USP7 levels (Figure 2B), suggesting that SCF^Cyclin F^ does not regulate USP7.

Additionally, we asked whether other treatments known to decrease or increase cyclin F levels would perturb USP7 protein levels. In agreement with previous reports, we found that cyclin F is downregulated in response to treatment with DNA-damaging agent, etoposide (Figure 2C). This effect was specific to cyclin F, since cyclin A2, with which cyclin F shares the highest amino acid sequence similarity, remained unperturbed (Figure 2C). In contrast, treatment of cells with a proteasomal inhibitor, MG132, resulted in buildup of a slower-migrating cyclin F band (Figure 2C). This slower-migrating species could be interpreted as ubiquitylated cyclin F; alternatively, it might represent phosphorylated form of cyclin F. Of note, SCF^β-TrCP^ has been shown to target cyclin F for ubiquitin-mediated degradation following its phosphorylation by casein kinase II [18]. Thus, the slower-migrating cyclin F band might be phosphorylated form of cyclin F, accumulating due to inhibition of its degradation post MG132 treatment. p21, a short-lived protein whose stability is also regulated by the ubiquitylation pathway, is also upregulated in response to MG132 treatment, indicating that an effective concentration of MG132 was used in our experiment (Figure 2C). Importantly, treatment with either etoposide or MG132 did not affect USP7 levels (Figure 2C). Collectively, these data suggest that SCF^Cyclin F^ does not regulate USP7 protein levels.

### Cyclin F protein and mRNA are downregulated in response to P22077 treatment

**Figure 3:**
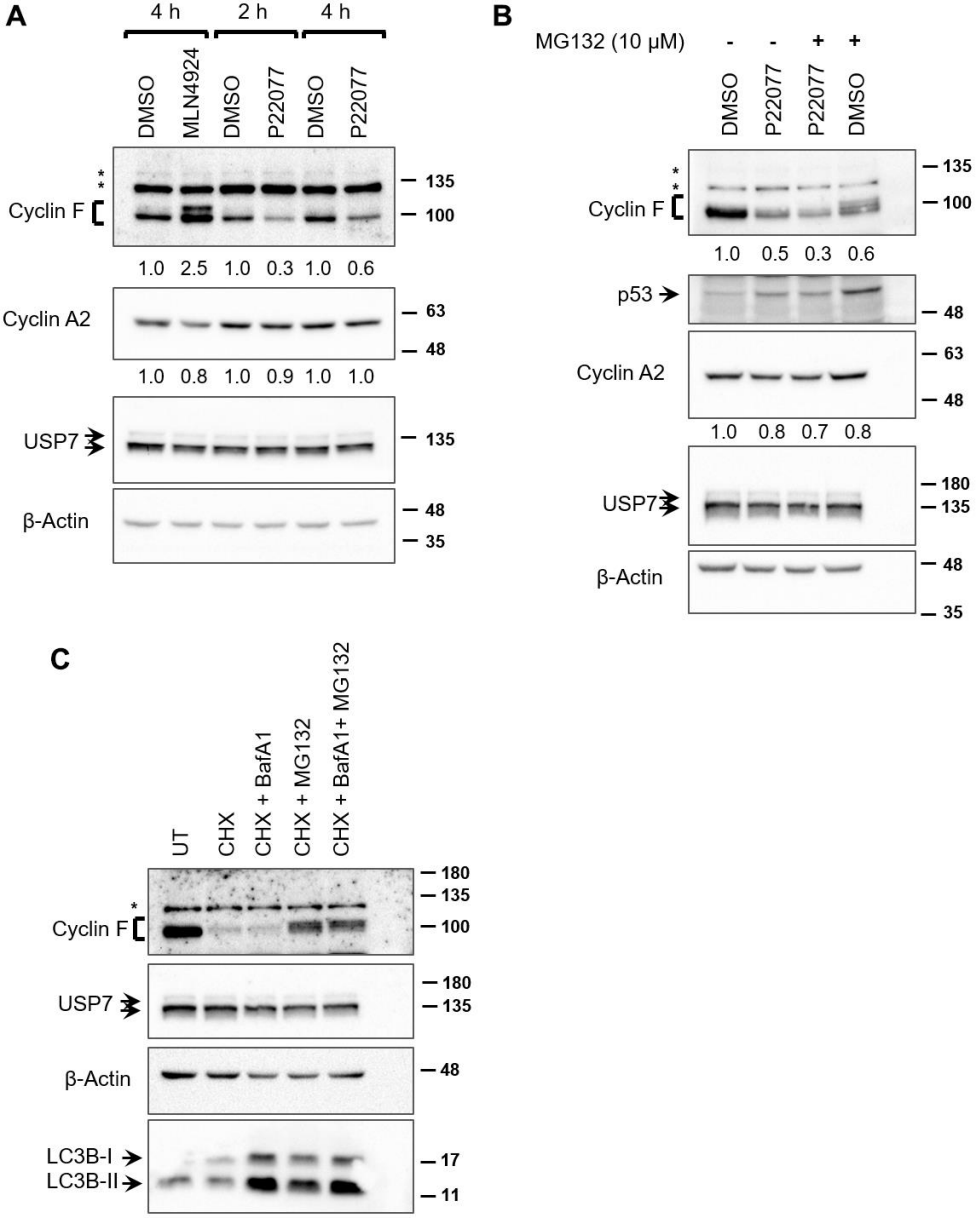
**USP7 regulates cyclin F protein levels.** (**A**) HeLa cells treated with DMSO, MLN4924 (10 μM), or P22077 (25 μM) for the indicated hours were lysed, and immunoblotted as indicated. β-Actin was the loading control. Asterisks denote non-specific bands. Fold changes were calculated with densitometric values for cyclin F and cyclin A2 blots using β-Actin as loading control. (**B**) HCT116 cells were treated with DMSO or P22077 (25 μM) for 5 h. Where indicated, cells were treated with MG132 (10 μM) for 4 h prior to harvest. Cells were then lysed and immunoblotted as indicated. Asterisks denote non-specific bands. Fold changes were calculated with densitometric values for cyclin F and cyclin A2 blots using β-Actin as loading control. (**C**) HCT116 cells were treated with DMSO or cycloheximide (CHX; 50 μg/mL) for 75 min. Where indicated, bafilomycin A1 (BafA1; 100 nM) and/or MG132 (12.5 μM) were added along with CHX. Cells were lysed, and immunoblotted as indicated. Asterisks denote non-specific bands.

We next sought to evaluate whether the deubiquitylase activity of USP7 contributed to regulation of cyclin F protein levels. We utilized the small-molecule inhibitor, P22077 which predominantly inhibits USP7 *in vivo* [24]. P22077 irreversibly inhibits USP7 by selectively forming a covalent bond with the critical Cys^223^ within the active site in the catalytic domain [25]. In unstressed cells, USP7 is required for the stability of MDM2, an E3 ubiquitin ligase responsible for ubiquitylating p53 and targeting it for proteasomal degradation [22, 26]. In USP7-depleted cells, auto-ubiquitylated-MDM2 becomes unstable, resulting in the stabilization of p53 [27]. Therefore, we sought to first confirm the inhibition of USP7 in our experiments by evaluating p53 levels in P22077-treated cells. HCT116 cells treated with P22077 showed an upregulation in p53 levels, indicative of USP7 inhibition (Figure 3B). We did not evaluate p53 levels in P22077-treated HeLa cells, where p53 is preferentially degraded by viral E6-dependent ubiquitylation [22]. Treatment of both HeLa and HCT116 cells with P22077 led to an approx. 70% reduction in cyclin F levels, as early as 2 h, compared to DMSO-treated controls (Figure 3A, 3B). On the other hand, cyclin A2, remained unperturbed by P22077 treatment (Figure 3A, 3B). These latter observations suggest that P22077 does not induce gross changes in cell cycle within the 4 h treatment timeframe, and that the reduction in cyclin F observed are likely a consequence of USP7 inhibition. Likewise, USP7 levels remained unaffected by P22077 treatment (Figure 3A, 3B). These data suggest the deubiquitylase activity of USP7 specifically plays a role in regulating cyclin F levels. Similarly, treatment of HCT116 cells with FT671, a highly specific inhibitor of USP7 and structurally unrelated to P22077, also led to approx. 70% reduction in cyclin F levels, compared to DMSO-treated control (Supplementary Figure 1) [28].

**Figure 4:**
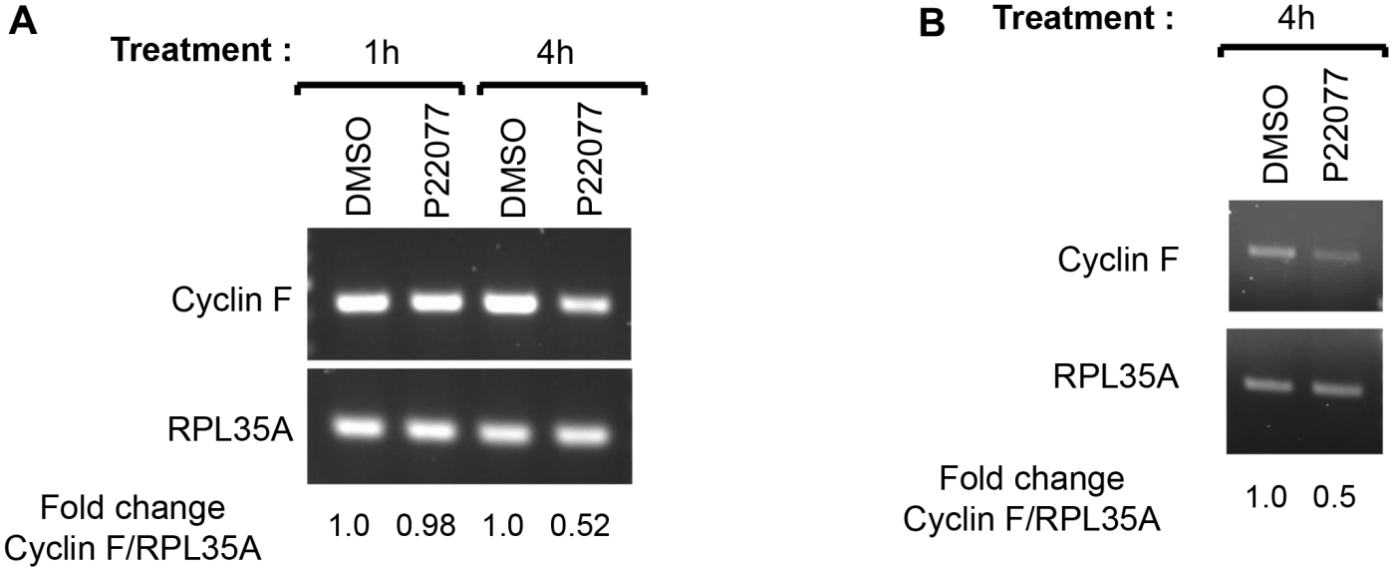
**USP7 regulates cyclin F mRNA levels.** (**A**) HeLa cells treated with DMSO or P22077 (25 μM) for the indicated hours were collected and total RNA was extracted. cDNAs prepared from each sample were amplified using primer pairs specific to each gene as indicated. The PCR products were analyzed by agarose gel electrophoresis and stained using ethidium bromide. RPL35A was the loading control. Fold change in cyclin F:RPL35A ratios were determined using densitometry values of the amplicons. The values represent an average from two independent experiments. (**B**) HCT116 cells treated with DMSO or P22077 (25 μM) for 4 h were analyzed as in (**A**).

We next asked whether inhibition of the proteasomes by MG132 treatment would rescue the P22077-mediated downregulation of cyclin F. Treatment with MG132 alone led to an accumulation of diffuse slower-migrating bands of cyclin F, compared to that in DMSO-treated control, suggestive of accumulation of ubiquitylated cyclin F as a consequence of proteasomal inhibition (Figure 3B). Treatment with MG132 could not rescue P22077-mediated reduction in cyclin F (Figure 3B). This observation prompted us to examine two possible scenarios: first, we ascertained whether cyclin F is indeed degraded by the ubiquitin-proteasome system as reported by others. We examined the stability of cyclin F using cycloheximide in the presence or absence of a pair of inhibitors, namely, MG132 or bafilomycin A1 (an inhibitor of vacuolar-type H(+)-ATPase and consequently an inhibitor of protein degradation in the lysosomes) [29]. Cyclin F levels robustly reduced to less than 50% compared to control, when *de novo* protein synthesis was inhibited by cycloheximide (Figure 3C). This reduction was rescued only when cells were co-treated with MG132, but not bafilomycin A1, suggesting cyclin F is indeed targeted for degradation by the proteasomal system and not by lysosomal degradation (Figure 3C). The accumulation of LC3B-II, which is degraded in the lysosomes, indicates that lysosomal enzymes were effectively inhibited at the concentration of bafilomycin A1 used (Figure 3C) [30]. Second, we examined whether the P22077-induced reduction in cyclin F protein levels was due to a reduction in cyclin F mRNA levels. We performed reverse transcriptase-PCR analysis on HeLa cells treated with DMSO or P22077, for 1 and 4 h. While there was no appreciable difference in cyclin F mRNA levels at 1 h between control- and P22077-treated cells, there was nearly 50% reduction at 4 h upon P22077 treatment (Figure 4A). These latter results suggest USP7 regulates cyclin F mRNA levels and that this regulation is dependent on its deubiquitylase activity. Similar effects were observed in HCT116 cells as well (Figure 4B).

### USP7 regulates cyclin F protein stability

**Figure 5:**
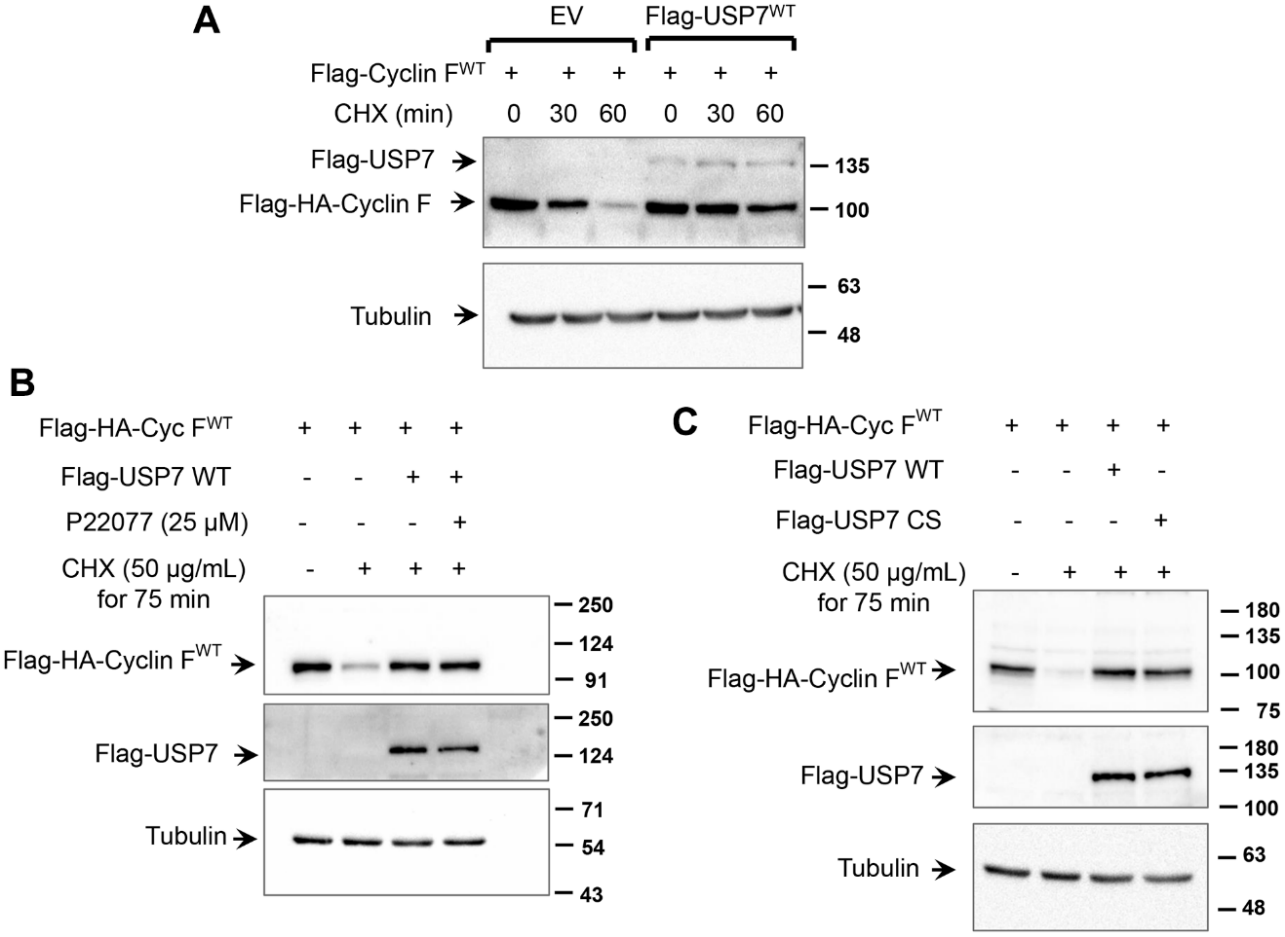
**USP7 regulates cyclin F protein stability.** (**A**) HCT116 cells were co-transfected with Flag-HA-Cyclin F WT and Flag-USP7 WT or empty vector. Twenty-four hours post transfection, cells were treated with DMSO or cycloheximide (CHX; 50 μg/mL) for the indicated minutes, lysed, and immunoblotted as indicated. α-Tubulin was the loading control. (**B**) HeLa cells were co-transfected with Flag-HA-Cyclin F WT and Flag-USP7 WT or empty vector. Forty-eight hours post transfection, cells were treated with CHX (50 μg/mL) for 75 min. Where indicated, P22077 (25 μM) was added to cells 2 h prior to the addition of CHX. Cells were then lysed, and immunoblotted as indicated. (**C**) HeLa cells were co-transfected with Flag-HA-Cyclin F WT and Flag-USP7 WT, Flag-USP7 CS, or empty vector. Forty-eight hours post transfection, cells were treated with CHX (50 μg/mL) for 75 min, lysed, and immunoblotted as indicated.

We next investigated whether USP7 regulates cyclin F protein stability. HCT116 cells were transfected with Flag-HA-cyclin F in the presence or absence of Flag-USP7-WT. As shown in Figure 5A, half-life of cyclin F was increased by USP7 overexpression (~ 60 min), whereas half-life of cyclin F in mock-transfected cells was about 30 min. We next evaluated whether this stabilization was dependent on the deubiquitylase activity of USP7. We employed two approaches: the first involved pharmacological inhibition of USP7 activity by P22077, and the second involved the expression of a catalytically-deficient mutant of USP7. Ectopic cyclin F levels robustly reduced to less than 50% of control levels, when *de novo* protein synthesis was inhibited by cycloheximide. This reduction was rescued in cells overexpressing wild-type Flag-USP7 (Figure 5B, 5C), and regardless of USP7 inhibition by P22077 (Figure 5B; last lane). These data suggest that the USP7-induced stability of cyclin F was independent of the deubiquitylase activity of USP7. This finding was further corroborated in experiments with the catalytic mutant of USP7, Flag-USP7-CS, that harbors a mutation of the critical Cys^223^ to Ser within the catalytic domain. As shown in Figure 5C, the cycloheximide-induced reduction in cyclin F levels was efficiently rescued in cells overexpressing Flag-USP7-CS, similar to that in cells overexpressing Flag-USP7-WT.

### Association between USP7 and cyclin F under genotoxic stress

**Figure 6:**
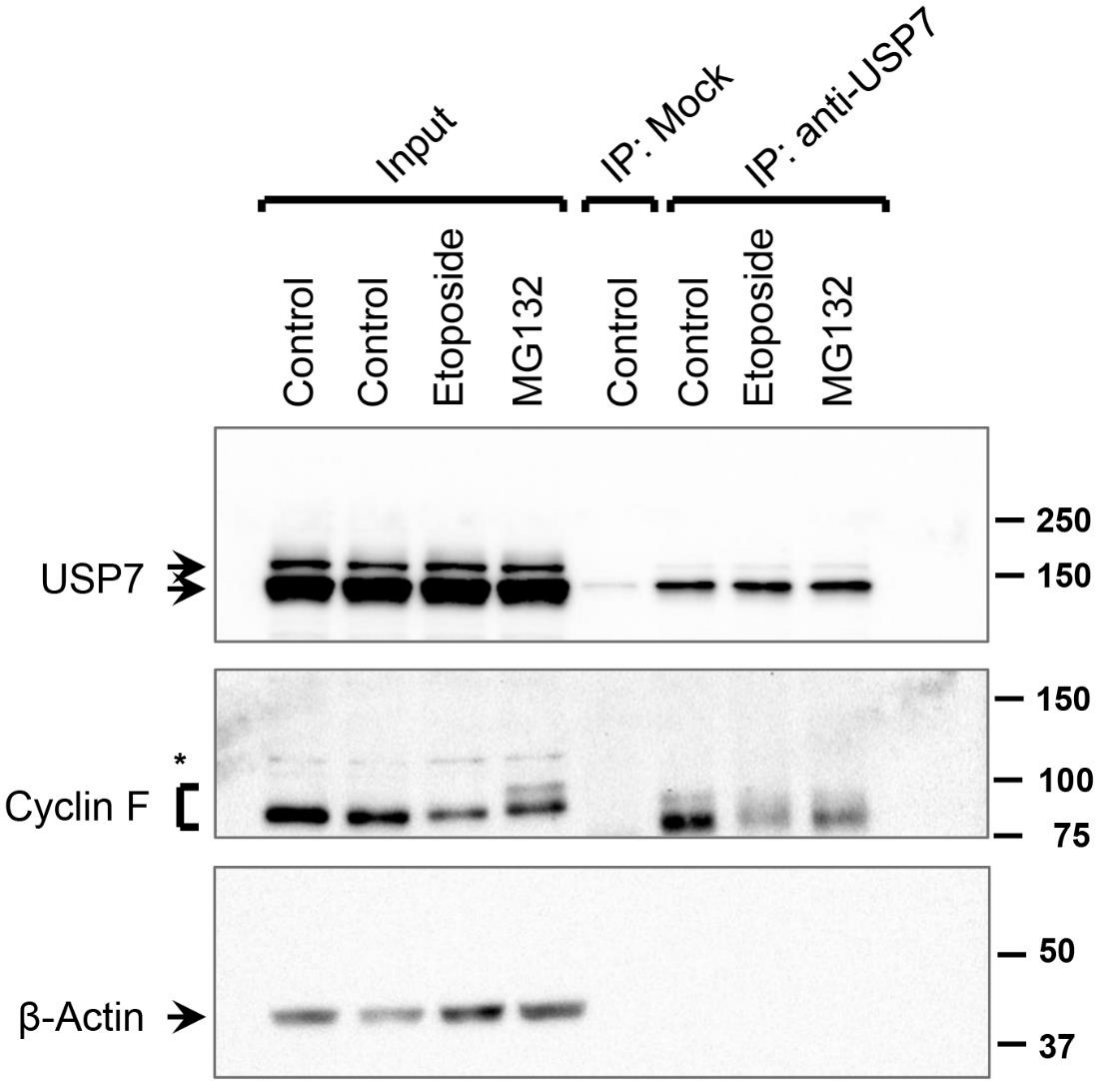
**Interaction between USP7 and cyclin F under DNA-damaging condition.** HeLa cells were treated with DMSO (control), etoposide (10 μM), or MG132 (10 μM) for 4 h. Endogenous USP7 was immunoprecipitated (IP) with anti-USP7 antibody or an unrelated, anti-p-TAK1 antibody (mock IP) as a loading control. Immunocomplexes were immunoblotted as indicated; β-actin was the loading control. Asterisk denotes non-specific band.

Consistent with previous reports, we observed that cyclin F protein levels are downregulated in cells treated with DNA-damaging agents, such as etoposide (Figure 2C). This downregulation has been attributed, in part, to increased proteasomal degradation of cyclin F following its ATR-dependent ubiquitylation [8]. We, therefore, hypothesized that under conditions of DNA damage, the interaction between cyclin F and USP7 might be reduced in order to afford a robust downregulation of cyclin F levels. To assess how the interaction between endogenous cyclin F and USP7 was influenced under unstressed and DNA-damaging conditions, we performed immunoprecipitation of endogenous USP7 from extracts of HeLa cells treated with or without etoposide. Western blot analysis showed that cyclin F was present in the anti-USP7 immunoprecipitates, but not in control immunoprecipitates with an unrelated, anti-p-TAK1 antibody (Figure 6). Notably, this association was weakly detected in cells subjected to genotoxic stress with etoposide, compared to that in untreated or MG132-treated cells (Figure 6). While the levels of cyclin F detected in the anti-USP7 immunoprecipitates only mirrors the cyclin F level present in the corresponding total extract (input lanes for Figure 6), it is also plausible that these results suggest that the association between USP7 and cyclin F might be disrupted in cells in response to DNA damage.

## DISCUSSION

Tight regulation of cyclin F during cell-cycle progression is key to avoiding unscheduled degradation of cyclin F substrates and preserving genomic integrity. To date, however, only a few interactors of cyclin F are known, and the molecular mechanisms regulating cyclin F abundance and SCF^Cyclin F^ ubiquitin ligase activity remain scantily understood. Cyclin F has a half-life of less than 1 hour [3]. It was initially suggested to undergo a metalloprotease-mediated degradation [20]; however, recent evidence indicate that it also degraded by the ubiquitin-proteasome system. Multiple E3 ubiquitin ligases that target cyclin F have been identified; APC/C^Cdh1^ targets cyclin F in the G1 phase, SCF^βTrCP^ during M phase, and an as yet unidentified, ATR-dependent E3 ubiquitin ligase targets it in response to genotoxic stress [8, 18, 21]. The identification of cyclin F-targeting E3 ubiquitin ligases suggest that there could be specific deubiquitylases that function to reverse the ubiquitylation on cyclin F and further fine-tune its abundance and/or activity.

In this study, we identify USP7 as a novel cyclin F-interacting protein and uncover novel aspects of cyclin F regulation mediated by this interaction. USP7 belongs to the ubiquitin-specific protease family of deubiquitylating enzymes, and interacts with a wide array of proteins to alter their stability, localization, and/or function [31]. While a large number of these interactors are regulated through the deubiquitylating activity of USP7, others are not direct targets, but are known to form stable complexes with USP7 [32]. We show that cyclin F associates with USP7 *in vivo*. A truncation mutant of cyclin F, harboring amino acids 1-270, also associates with USP7, suggesting that the cyclin-box and PEST regions are dispensable for this interaction. Of note, the N-terminal region of USP7 harbors a TRAF-domain that recognizes a consensus TRAF-recognition motif, P/A/E-x-x-S, present in many USP7-interacting proteins [31]. The N-terminal 1-270 region of cyclin F harbors multiple putative consensus TRAF-recognition motifs. It remains to be determined whether any of these motifs are crucial for the interaction between cyclin F and USP7.

A surprisingly large number of USP7 interactors are E3 ubiquitin ligases [32]. For example, interaction between USP7 and MDM2 results in USP7-mediated deubiquitylation of MDM2 and enhanced stability of the latter [22, 26]. On the other hand, the interaction between USP7 and TRIM27 (another E3 ubiquitin ligase) results in multiple outcomes: USP7 not only deubiquitylates TRIM27, but the latter in turn, binds to and ubiquitylates USP7, which facilitates recruitment and deubiquitylation of another protein, RIP1 by USP7 [33]. To uncover the functional consequences of cyclin F-USP7 interaction, we first investigated how the two proteins influence each other. Our data suggest that USP7 associates with cyclin F to regulate cyclin F abundance and stability, and not *vice versa*. First, neither forced expression of cyclin F, nor inhibition of the SCF^Cyclin F^ activity, affected USP7 protein levels. Second, ectopic expression of USP7 increased the protein stability of ectopic cyclin F. To our surprise, however, the USP7-induced stability of cyclin F protein was independent of the deubiquitylase activity of USP7. Wild-type USP7 was able to stabilize cyclin F even when the deubiquitylase activity of USP7 was inhibited by P22077. Likewise, a catalytically-inactive mutant of USP7 was also able to stabilize cyclin F protein levels, similar to wild-type protein. Together, these observations suggest that USP7 regulates cyclin F protein stability indirectly, presumably, by either blocking autoubiquitylation of cyclin F by SCF^Cyclin F^ or by sequestering cyclin F away from another E3 ubiquitin ligase or non-lysosomal protease. Of note, our data reveal that lysosomal proteases do not target cyclin F for degradation.

In contrast to the overexpression studies mentioned above where USP7 stabilizes cyclin F in a deubiquitylase-activity-independent manner, treatment of asynchronously growing cells with P22077 or FT671 resulted in a rapid downregulation of endogenous cyclin F protein levels. These latter observations suggest a deubiquitylase-activity-dependent regulation of cyclin F abundance by USP7. Our data suggest that this deubiquitylase-activity-dependent effect on cyclin F protein levels likely stems from the regulation of cyclin F mRNA by USP7: pharmacological inhibition of USP7 by P22077 downregulated cyclin F mRNA levels. USP7 is known to regulate activity of many transcription factors. Hence, it is conceivable that USP7 regulates the activity of a specific transcription factor or coregulator involved in the transcriptional control of cyclin F. Alternatively, USP7 may regulate factors that are important for the stability of cyclin F mRNA. Collectively, it is noteworthy that USP7 exerts both deubiquitylase-activity–dependent as well as –independent actions to regulate cyclin F abundance.

In many instances, USP7 has been found to switch from E3 ubiquitin ligase to its substrate in response to cellular stress [32]. A prime example of this molecular switch is seen in the regulation of the MDM2-p53 axis by USP7. Under normal conditions, USP7 interacts with and stabilizes MDM2, resulting in enhanced ubiquitin-mediated degradation of its target, p53 [22, 26]. Upon DNA damage, however, USP7 switches from MDM2 to p53, resulting in deubiquitylation and stabilization of the latter [27]. We hypothesize that USP7 might operate, similarly, as a molecular switch in the regulation of cyclin F and consequently, its substrate(s). Further studies are warranted to investigate the functional interaction between cyclin F and USP7 in normal cell-cycle control and conditions of genotoxic stress.

Our data predict that USP7-inhibited or –depleted cells are likely to express low levels of cyclin F and exhibit centrosome overamplification and genomic instability. Of note, depletion of USP7 results in overamplified centrosomes, multipolar spindles, chromosome misalignment, and cytokinetic defects in multiple cell lines, by directly or indirectly affecting the stability of many key proteins [34–36]. Whether reduction in cyclin F levels contributes to any of these USP7 depletion-induced phenotypes remains to be investigated. USP7 expression is frequently misregulated in various cancer types and has context-dependent tumor suppressor or oncogenic roles [31]. Likewise, USP7 loss-of-function mutations have been detected in pediatric leukemias [37]. While initial evidence suggests a tumor-suppressor function for cyclin F, emerging reports indicate that it may also have a context-dependent oncogenic function in certain cancers [38]. Thus, it would be clinically relevant to investigate whether the USP7-cyclin F axis contributes to tumor progression and aggressiveness in these specific contexts.

In conclusion, in this study, we demonstrate a new interacting partner of cyclin F, namely USP7, and the role of USP7 in the regulation of cyclin F mRNA and protein. This study highlights a potential role for the cyclin F-USP7 axis in pathological conditions, including cancer and neurodegenerative diseases.

## MATERIALS AND METHODS

### Expression vectors

Flag-HA-tagged Cyclin F construct was a kind gift from Dr. Michele Pagano (NYU Grossman School of Medicine, USA) [15]. Flag-HA-Cyclin F^1-270^ (truncation mutant) was created by site-directed mutagenesis using Flag-HA-Cyclin F wild-type construct as the template. Presence of the desired mutation was verified by sequencing. pQFlag-USP7-WT and pQFlag-USP7-CS were purchased from Addgene (plasmids #46751 and #46752, respectively, deposited by Drs. Goedele Maertens and Gordon Peters).

### Cell culture and transfections

HEK-293T, HeLa, and HCT116 cell lines were cultured in DMEM (Sigma-Aldrich, MO, USA) containing 10% fetal bovine serum (Thermo Fisher Scientific, MA, USA) supplemented with 100 IU/mL penicillin and 100 mg/mL streptomycin (Thermo Fisher Scientific). All cell lines were maintained in a humidified incubator at 37° C with 5% CO_2_. Where indicated, cells were transfected with expression vectors using Lipofectamine 3000 (Thermo Fisher Scientific) according to manufacturer’s instructions.

### Chemical inhibitors

Protease inhibitor cocktail set III and phosphatase inhibitor cocktail set V were purchased from Sigma-Aldrich. Where indicated, cells were treated with 10 or 12.5 μM MG132 (Sigma-Aldrich), 3 μM MLN4924 (Sigma-Aldrich), 25 μM P22077 (Sigma-Aldrich), 10 μM FT671 (MedChemExpress, NJ, USA), and 100 nM Bafilomycin A1 (Sigma-Aldrich).

### RNA isolation and reverse transcriptase-PCR

Total RNA was purified from HeLa and HCT116 cells using TRI reagent (Sigma-Aldrich) according to manufacturer’s instructions. Single-stranded cDNA was reverse transcribed from 1 μg of total RNA using high-capacity cDNA reverse transcription kit (Thermo Fisher Scientific) according to manufacturer’s instruction. One microliter of the RT reaction product was mixed with 1x DreamTaq PCR master mix (Thermo Fisher Scientific) and 0.5 μM of each primer in a total reaction volume of 20 μL. The primers used were as follows: 1) human cyclin F: forward – ^5’^CCAGGGAACCTGAAGCTCTTT^3’^ and reverse – ^5’^TCGCTTTCCCAGAGGAGGTA^3’^: 2) human RPL35A: forward – ^5'^GGGTACAGCATCACTCGGA^3'^ and reverse – ^5'^ACGCCCGAGATGAAACAG^3'^. PCR cycles were carried out using the Eppendorf Mastercycler Nexus GX2. The PCR products were analyzed by electrophoresis in 1 % agarose gels and visualized by staining with ethidium bromide. RPL35A expression was used for normalization.

### Immunoprecipitation and immunoblotting

For immunoprecipitations, extracts were prepared by cell lysis in immunoprecipitation buffer (50 mM HEPES, pH 7.2; 150 mM NaCl; 1 % NP-40; 1 mM EDTA; 1:100 dilution of protease inhibitor cocktail set III; 1:50 dilution of phosphatase inhibitor cocktail set V; and 0.5 mM dithiothreitol). Cleared lysates were incubated with antibody for 16 h, and subsequently with protein A/G-agarose beads (Thermo Fisher Scientific) for 2 h, at 4° C, on a rotator. Immunocomplexes were washed 3 times with immunoprecipitation buffer and eluted bound-proteins were resolved in 8 or 12 % SDS-polyacrylamide gels. For other experiments, extracts were prepared by cell lysis in RIPA buffer (50 mM HEPES, pH 7.2; 150 mM NaCl; 1 % NP-40; 0.5 % sodium deoxycholate; 0.1 % SDS; 1 mM EDTA; 1:100 dilution of protease inhibitor cocktail set III, 1 mM orthovanadate; 5 mM NaF; and 1 mM dithiothreitol). Following electrophoresis, proteins were transferred onto a PVDF membrane. Blocking and incubation with primary antibodies were performed in Tris-buffered saline containing 0.1 % Tween-20 and supplemented with 5 % skimmed milk powder. Proteins were visualized by ChemiDoc XRC+ gel imaging system (Bio-Rad, CA, USA), using secondary horseradish peroxidase-conjugated antibodies and enhanced chemiluminescence reagent (femtoLucent plus-HRP, G-Biosciences, MO, USA).

### Antibody sources

Antibodies to cyclin F (D9K2U), USP7 (D17C6), UBR5 (D6O8z), LC3 (4775S), and β-Actin (13E5) were from Cell Signaling Technology (MA, USA). Anti-HA-probe (F7) was from Santa Cruz (TX, USA), anti-cyclin A2 (BF683) from Bethyl Laboratories (TX, USA), anti-p53 (DO7) from Abcam (Cambridge, UK), anti-rabbit and anti-mouse IgG-HRP from Genei Laboratories (Bangalore, India), and anti-rabbit IgG-HRP from Cloud-Clone Corp (TX, USA).

### Data availability statement

The authors confirm that the data supporting the findings of this study are available within the article.

## Supplementary Material

Supplementary Information

Supplementary Figure 1

Supplementary File 1
