# Supplementary material for: The deubiquitylase USP7 is a novel cyclin F-interacting protein and regulates cyclin F protein stability
Source: Aging (Albany NY). 2022 Nov 5;14(21):8645–60. doi: 10.18632/aging.204372 (PMC9699750; doi:10.18632/aging.204372)
Supplement: Supplementary Information [file aging-14-204372-s001.pdf]

SUPPLEMENTARY INFORMATION

Supplementary experimental procedures

Affinity purification and tandem mass spectrometry

We transiently expressed Flag-HA-Cyclin F or empty vector (as negative control) in cells from the U2OS human osteosarcoma cell line. 24 h post-transfection, we synchronized cells in prometaphase using a nocodazole (100 ng/ml) block. Cells were then released from the block into fresh medium, collected at 8 and 16 h time points. Whole-cell extracts were prepared by lysing cells for 45 min in immunoprecipitation buffer (50 mM HEPES, pH 7.2, 150 mM NaCl, 1.0 % nonyl phenoxypolyethoxylethanol-40 (NP-40), 1 mM ethylenediamine tetraacetic acid, 1 mM ethyleneglycol tetraacetic acid, 1:100 dilution of protease inhibitor cocktail set III, 1:100 dilution of phosphatase inhibitor cocktail set I, 1 mM orthovanadate, 1 mM NaF, 0.5 mM dithiothreitol). Cleared lysates were incubated with anti-HA antibody-conjugated resin (Sigma-Aldrich, St Louis, MO, USA) directed against the HA-tag, for 16 h. Immune complexes were washed 5 x with immuno-precipitation buffer. The immunoprecipitates were eluted from the beads with 100 µg/ml of HA-peptide (Sigma-Aldrich).

The eluate was processed using the expertise of the proteomics core facility at the Moffitt Cancer Center & Research Institute, USA. Briefly, the eluate were then resolved on a 10% SDS-PAGE gel and visualized by Coomassie Brilliant Blue staining. Each lane was cut into 10 equal slices. To reduce and alkylate proteins, destained-gel slices were treated with TCEP (Tris (2-carboxyethyl) phosphine and iodoacetamide. Following in-gel tryptic digestion, peptides were extracted and concentrated under vacuum centrifugation. A nanoflow ultra high performance liquid chromatograph (RSLC, Dionex, Sunnyvale, CA, USA) coupled to an electrospray ion trap mass spectrometer (LTQ-Orbitrap, Thermo, San Jose, CA, USA) was used for tandem mass spectrometry peptide sequencing experiments. The sample was first loaded onto a pre-column (2 cm x 75 µm ID packed with C18 reversed-phase resin, 5 µm, 100 Å) and washed for 8 minutes with aqueous 2 % acetonitrile and 0.04 % trifluoroacetic acid. The trapped peptides were eluted onto the analytical column, (C18, 75 µm ID x 50 cm, Pepmap 100, Dionex, Sunnyvale, CA, USA). The 120-minute gradient was programmed as: 95% solvent A (2% acetonitrile + 0.1% formic acid) for 8 minutes, solvent B (90% acetonitrile + 0.1% formic acid) from 5% to 15% in 5 minutes, 15% to 40% in 85 minutes, then solvent B from 50% to 90% B in 7 minutes and held at 90% for 5 minutes, followed by solvent B from 90% to 5% in 1 minute and re-equilibrate for 10 minutes. The flow rate on analytical column was 300 nl/min. Five tandem mass spectra were collected in a data-dependent manner following each survey scan. The MS scans were performed in Orbitrap to obtain accurate peptide mass measurement and the MS/MS scans were performed in linear ion trap using 60 second exclusion for previously sampled peptide peaks. Mascot searches were performed against the Swiss-Prot human database downloaded on June 11, 2014. Two trypsin missed cleavages were allowed, the precursor mass tolerance was 1.08 Da. MS/MS mass tolerance was 0.8 Da. Dynamic modifications included carbamidomethylation (Cys), oxidation (Met), and phosphorylation (Ser/Thr/Tyr). Results from Mascot were compiled in Scaffold, which was used for manual inspection of peptide assignments and protein identifications. Using these, we have further compiled a list of putative cyclin F-associated proteins (provided as an Excel file; see Supplementary File 1) that were identified based on the presence of at least two unique peptides with a minimum peptide probability threshold of 95 % and minimum protein probability threshold of 95% (Supplementary File 1).
