# Supplementary material for: The deubiquitylase USP7 is a novel cyclin F-interacting protein and regulates cyclin F protein stability
Source: Aging (Albany NY). 2022 Nov 5;14(21):8645–60. doi: 10.18632/aging.204372 (PMC9699750; doi:10.18632/aging.204372)
Supplement: Supplementary Figure 1 [file aging-14-204372-s002.pdf]

SUPPLEMENTARY FIGURE

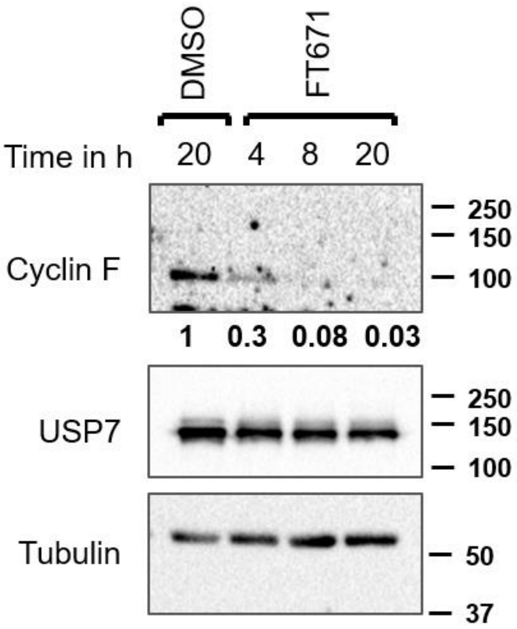

Supplementary Figure 1. USP7 regulates cyclin F protein levels. HCT116 cells treated with DMSO or FT671 (10 μM) for the indicated hours were lysed, and immunoblotted as indicated. β-Actin was the loading control. Asterisks denote non-specific bands. Fold changes were calculated with densitometric values for cyclin F blot using tubulin as loading control.
